# Brief Autism Mealtime Behavior Inventory (BAMBI): Italian Translation and Validation

**DOI:** 10.3390/children10071201

**Published:** 2023-07-11

**Authors:** Antonella Lamboglia, Roberta Romano, Donatella Valente, Anna Berardi, Gioia Cavalli, Federica Giovannone, Carla Sogos, Marco Tofani, Giovanni Galeoto

**Affiliations:** 1 MSC in Rehabilitation Sciences for Health Professions, Sapienza University of Rome, P.le Aldo Moro 5, 00185 Rome, Italy; lamboglia.1916093@studenti.uniroma1.it (A.L.); romano.1701035@studenti.uniroma1.it (R.R.); 2 Department of Human Neurosciences, Sapienza University of Rome, Viale dell’Università 30, 00185 Rome, Italy; donatella.valente@uniroma1.it (D.V.); anna.berardi@uniroma1.it (A.B.); gioia.cavalli@uniroma1.it (G.C.); federica.giovannone@uniroma1.it (F.G.); carla.sogos@uniroma1.it (C.S.); marco.tofani@uniroma1.it (M.T.); 3 Neuromed IRCCS, 86077 Pozzili, Italy

**Keywords:** autism spectrum disorder, feeding, food selectivity, behavior, mealtime, children, BAMBI, scale, psychometric properties, reliability

## Abstract

Food selectivity is among the most common problems for children with Autism Spectrum Disorder (ASD). The present study aims to validate the Brief Autism Mealtime Behavior Inventory (BAMBI) in an Italian population of children with ASD. BAMBI was translated and cross-culturally adapted following international guidelines, then we investigated internal consistency as measured by Cronbach’s alpha and test–retest reliability, as measured by the Intraclass Correlation Coefficient (ICC) in a sample of both children with ASD and with typical development (TD). A total of 131 children were recruited in a clinical and community sample. Internal consistency revealed significant data for both TD and ASD children, with a Cronbach’s Alpha of 0.86 and 0.71, respectively. Test–retest reliability showed excellent values for each item of the BAMBI (range 0.83–1.00). Furthermore, we investigated differences in gender and body max index; however, no significant differences were found among groups. In conclusion, the Italian version of the BAMBI showed good internal consistency and test–retest reliability and it can be used for clinical and research purposes.

## 1. Introduction

Autism Spectrum Disorder (ASD) is an early onset neurodevelopmental disorder primarily affecting two main areas: ‘social communication’, and ‘restricted, repetitive and/or sensory behaviors or interests’ [1]. It affects about 1 in every 150 children, regardless of ethnic and social groups, with a higher prevalence in males than females at a ratio of 4:1 [2]. In Italy, it is estimated that 1 in 77 children (aged 7–9 years old) is diagnosed with ASD, with a prevalence 4.4 times higher among boys than among girls [3,4].

Feeding and eating problems are common problems that affect persons with ASD across all ages and cognitive abilities. It is well known that children with typical development, especially at preschool age, show an attitude of preference or rejection toward some foods; in these cases, children are referred to as “picky eaters”. Scientific literature points out that this behavior declines around the age of 6 years, when children have more opportunities to eat outside the family context and are exposed to a greater variety of foods that promotes the extinction of dietary restrictions [5]. In the population with ASD, food selectivity may be present at a very early age and may be persistent during the life course. Food selectivity is not a valid diagnostic criterion, but this aspect is emphasized in the DSM 5 as a defining characteristic of ASD [6]. Food selectivity appears to be the eating behavior most frequently associated with ASD, which is present in more than 70 percent of the pediatric population with ASD [7]. The term food selectivity is used to describe a wide range of eating situations or behaviors that are engaged in during mealtimes, and this expression refers to the refusal of food, the presence of a limited repertoire of accepted foods, and the intake of a single food at a high frequency [8]. In this regard, rigid and repetitive behavior patterns are characteristic of ASD, and excessive adherence to the routine may underlie extreme restrictions on the types of food consumed [9]. Also, regarding the insistence on eating a narrow variety of foods, typically, the selection is made by the child based on sensory characteristics of the foods, such as taste, color, temperature, or texture [10]. In addition, very often children with ASD have gastrointestinal difficulties, such as constipation, vomiting, and food allergies. Children with ASD consume significantly more sugary drinks and snacks daily but fewer daily servings of fruits and vegetables [11]. In this regard, research has been conducted regarding the nutritional consequences of a restricted diet in individuals with ASD [12]; however, feeding problems do not necessarily translate into an increased risk of impaired growth.

Therefore, the assessment of feeding problems in ASD should be included in routine screenings, and it would be appropriate for health professionals to have a greater awareness of this topic [13]. Food selectivity can be assessed with different approaches: (a) direct observation of feeding behavior, (b) the use of specific assessment tools or (c) through a general assessment that includes the use of a food diary and swallowing analysis [14]. Several assessment tools are used for food selectivity: checklists, questionnaires, interviews and surveys completed by parents or caregivers [8]. However, a recent systematic review [15] strengthened the need of specific assessment tool to measure feeding and eating problems in children with ASD: most of the instruments were created for children with typical development and then applied to populations of children with ASD; in other cases, authors have developed their “own tools” without evaluating appropriate psychometric properties. To address this gap, the Brief Autism Mealtime Behavior Inventory (BAMBI) [16] was created. The BAMBI is the first tool explicitly developed to assess eating difficulties of children with ASD [17]. The initiative to develop a specific tool was crucial and contributed to the underlining of the importance of eating-related problems in the pediatric population. BAMBI is a measurement tool able to discriminate eating problems presented by individuals with ASD compared to those of children with typical development. BAMBI focuses on eating-related behaviors, disregarding the sensorial and gastrointestinal problems frequently found in ASD that can influence one’s eating behavior.

The need to have validated tools to measure a specific construct is well noted. Considering the lack of specific assessment tools on this topic, no measurement tools have been developed or adapted to measure the mealtime and feeding problems of individuals with ASD or other neurodevelopmental disorders in Italy; therefore, the present investigation aims to translate the BAMBI into Italian and to validate its psychometric properties in an Italian population of children with ASD. 

## 2. Methods

The study was conducted from June 2021 until September 2021. The present investigation was carried out by a research group of Italian rehabilitation and healthcare professionals within the Child Neuropsychiatry Unit of the Department of Human Neurosciences of Sapienza University of Rome, together with the collaboration of a non-profit organization R.O.M.A.—Rehabilitation and Outcome Measures Assessment. The research group has great experience with validation outcome measures in children [18,19,20,21,22].

### 2.1. Assessment Tool

BAMBI [16] is an assessment tool developed in English. The tool was validated on children aged 3–11 years with or without a diagnosis of ASD. The BAMBI is an observer-reported outcome measure specifically designed for parents and primary caregivers and it was designed to capture mealtime behaviors specific to children with ASD. The BAMBI is scored on a 5-point Likert scale, from a score of 1, indicating that a specific behavior “never” occurs, to a score of 5, indicating the behavior “always” occurs during mealtime. Reversed scoring is used for four of the items rating positive mealtime behaviors. A total frequency score is calculated from a sum of all 18 items with higher scores reflecting more mealtime behavior problems. An exploratory factor analysis identified three factors, namely Limited Variety, Food Refusal, and Features of Autism. The internal consistency showed a Cronbach’s alpha of 0.88 and ranged between the three factors (0.87 for limited variety, 0.76 for food refusal, and 0.63 for features of autism) [16,23]. Lukens and colleagues [16] found high values for both test–retest (r = 0.87) and inter-rater reliability between a parent and teacher or therapist report (r = 0.78). The Limited Variety factor demonstrated strong construct validity when correlated with intake of vegetables, as measured by the 24 h Recall Interview (r = 0.87). Besides the original version, BAMBI [16,23] was translated and validated into Portuguese-Brazilian [24], Turkish [25] and Vietnamese [26].

### 2.2. Translation and Cross-Cultural Adaptation

Following the recommendation of the Beaton and colleagues [27], the translation and cross-cultural adaptation process was completed in several phases. First, two forward translations of the original version of the BAMBI were independently produced by two speech and language therapists confident with both English and Italian. These translations were then synthesized into a single document by the study coordinator (GG). Then, two bilingual rehabilitation professionals independently translated back the BAMBI and submitted a written report highlighting any difficult or unclear phrases.

An expert committee, comprising all translators and the research group, reviewed the translated versions, and discussed discrepancies and difficulties during the process. This led towards a pre-final version of the BAMBI that was administered to the first five participants as a pre-test. There were interviewed and answered questions concerning the wording and comprehensibility of the tool. Consensus was achieved and the final Italian version of the BAMBI was produced (see Appendix A).

### 2.3. Participants

We recruited children aged between 6 and 10 years, with parent who demonstrated good ability to communicate in Italian. For the objective of the present study, we opted to include a convenience sample of both children with typical development and children with ASD. Children with a certified diagnoses of ASD were recruited in the outpatient clinic of the Institute of Child Neuropsychiatry of the Policlinico Umberto I University Hospital in Rome, while children with typical development were recruited in a primary school nearby the aforementioned institute. Participants with other neurodevelopmental disorders and genetic syndromes (i.e., Rett Syndrome or Fragile X Syndrome) were excluded. 

### 2.4. Procedures and Data Analysis

Before starting the study, the research group participated in an internal training course to increase confidence in administering BAMBI. A speech therapist with significant experience using the assessment tools led this training. 

The recruitment period lasted four months, from June to September 2021. Before administering the BAMBI, parents were asked about the socio-demographic information of the family (members, nationality, parents’ occupation) and information on the child, in particular age, weight, height, and the presence of gastric disorders or eating problems. Data for children with ASD were obtained from clinical records and parents’ interviews, while for children with typical development, information was collected by two speech and language therapists (AL, RR). Once inclusion and exclusion criteria were verified, the research team administered the BAMBI. 

Data were summarized and analyzed using frequency tables, means, and standard deviations. We used Cronbach’s Alpha to measure and investigate internal consistency. Cronbach’s Alpha expresses the correlations between different items on the same tool; a coefficient greater than 0.7 indicates acceptable internal consistency [28,29]. Another crucial property for reliability analysis is the test–retest reliability, indicating stability over the time of the tool. For test–retest reliability of BAMBI, we used two repeated measurements of the same participant by the same rater, 2 days apart. We selected 30 participants from the original sample according to their participation availability. To calculate reliability, we used the Intraclass Correlation Coefficients (ICC) with 95% confidence. Values for ICC range from 0 to 1.00 and can be interpreted as follows: 0.00–0.25, little, if any, correlation; 0.26–0.49, low correlation; 0.50–0.69, moderate correlation; 0.70–0.89, high correlation; and 0.90–1, very high correlation [29,30]. All statistical analyses were performed using IBM Statistical Package for the Social Sciences (SPSS, Version 20.0).

## 3. Results

We recruited 47 individuals with ASD and 90 individuals with typical development. Of these, six were excluded due to lack of consent to data processing. Therefore, the final sample was composed of 84 children with typical development with a mean age of 6.73 (SD 2.5) and 47 children with a diagnosis of ASD with a mean age of 7.45 (SD 1.8). Sample characteristics are summarized in Table 1. 

### 3.1. Internal Consistency

Internal consistency analysis showed statistical significance for both children with typical development and children with ASD, with a Cronbach’s Alpha of 0.86 and 0.71, respectively. To better understand differences and how items work in both groups, we reported the data of Items—total statistics. The findings are summarized in Table 2.

For test–retest reliability, we reported a descriptive analysis of mean (SD) scores of the two repeated measurements and ICC values with 95% CI, resulting in a range of 0.83–1.00. Table 3 synthesizes values for each item of BAMBI.

### 3.2. Differences among Body Mass Index (BMI) and Gender

**Figure 1:**
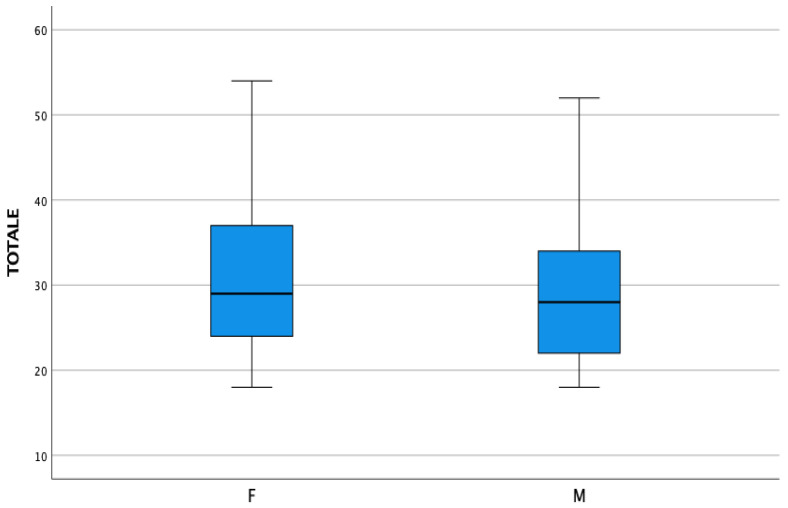
Differences in BAMBI across gender.

**Figure 2:**
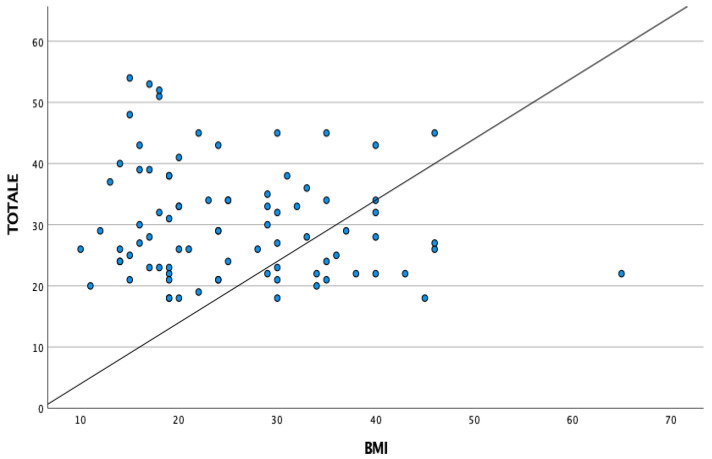
Distribution of BAMBI score and BMI.

We also investigate differences in mean scores among gender and BMI. From the analysis, we can assert that there are no differences in gender and correlation between BMI and total BAMBI score. Data are reported in Figure 1 and Figure 2.

## 4. Discussion

The present study aims to preliminarily validate the Italian version of the BAMBI in a sample of children with both ASD and with typical development. Recently, the DSM-V included diagnostic criteria for Avoidant and Restrictive Food Intake Disorder (ARFID) [31], characterized by limiting an individual’s food intake; although it is not a specific symptom for the ASD population, children with ASD were found to have slightly poorer self-feeding skills and were more likely to avoid foods and to exhibit food neophobic behaviors [32]. The BAMBI proved to be an ideal tool for this objective, as it includes three specific domains (Limited Variety, Food Refusal, Autism Characteristics) and effectively assesses the construct under investigation; it could therefore be integrated not only into clinical routines but could also target direct and indirect treatments on eating problems and possible consequences. Now available in Italian, this tool could also alert family members and professionals about feeding problems, even before a formal diagnosis. This is of utmost importance, since parents often report mealtimes as problematic. In fact, many children with ASD have feeding challenges due to their ASD symptomology and associated behavioral, cognitive, psychological, or familial factors [33].

One of the most frequently cited feeding problems for patients with ASD is the limited variety of food intake [34], which could be related to one of the clinical symptoms used to diagnose ASD: restricted behavior and interests [31]. The group of individuals with ASD has lower levels of eating different foods. This behavior, e.g., eating only a particular variety of foods, is the situation that recurs most often in this population, which moreover has a stronger preference for starches, snacks, processed foods, and an aversion to fruits, vegetables, and protein foods [35]. The limited variety of food consumed and food refusal usually occur together. Aspects related to food rejection could be related to texture, color, appearance, odor, and temperature [36,37,38]. Several studies confirmed a significantly higher frequency of food selectivity in children with ASD than in their typically developing peers [8,39,40]. In fact, sensory sensitivity may lead children with ASD to restrict their food choices, sticking to their preferred, tolerable, and manageable textures [41,42]. BAMBI allows us to measure specific features of dysfunctional habit during mealtimes, also investigating accepted food and texture. Food texture and taste are thought to drive food choices and impact the food acceptance of children with ASD, and children with ASD [43,44] frequently show many food dislikes, repetitive food choices, and resistance to new taste experiences [45,46,47].

The BAMBI questionnaire also allows for ASD characteristics to be assessed through some questions (3, 5, 6, 9, 18). Children with ASD, compared with controls, are found to be more aggressive at mealtimes, have difficulty remaining seated until the meal is finished, and exhibit less flexibility in mealtime routines [48]. Further studies report that eating problems in children with ASD do not correlate with the disorder’s severity level [49,50].

The BAMBI is a simple and quick-to-use Observer Reported Outcome, which is a measurement based on an observation by someone other than the patient or a health professional in general. The original version of the BAMBI was translated into Italian following international guidelines. Equivalence between the Italian version of the BAMBI with the original was investigated on a semantical domain. Only a few modifications were suggested; for example, Item 8 was literally translated using the Italian verb “chiudere” (close in English), but the research group opted to insert the synonym verb “serrare” because it indicated closing the mouth as a refusal behavior. Also, Item 18 was modified because the original version reported as an example “fried foods, cold cereals, raw vegetables”, while the research group opted to insert “pasta” because more appropriate for the Italian culture and eating habits of families; therefore, the examples of item 18 were modified as “cibi fritti, pasta fredda e verdure cruda” (fried foods, pasta, and raw vegetables). These changes were also discussed with participants during the pre-test phase. Participants’ observation allowed us to gain cross-cultural validity and proved to be strictly related to the meaning of the original items.

For internal consistency, as measured with Cronbach’s coefficient alpha, our findings showed significant values for both children with typical development (0.86) and children with ASD (0.71), in line with the Turkish version (α 0.79) [25], the Vietnamese (α 0.78) [26], the Brazilian (α 0.70) [24] and the original version (α 0.88). Our results also revealed good test–retest reliability, in line with Lukens’ study [16,23], while no other validation studies analyzed this issue and therefore it was not possible to perform any other comparison. However, test–retest reliability is an important psychometric property for determining the appropriateness of measurement tool [51], and BAMBI was seen as a reliable tool over time. Concerning item–total correlations, our results demonstrate that all items positively contribute to determining the construct of the BAMBI, as reported in other validation studies [16,23,26]. We also calculated if differences among sex/gender exist. Our results do not find significant differences between males and females. Wallace and colleagues [52] tried to verify whether sex was related to ASD-specific eating problems, but they did not reach robust conclusions, despite reporting qualitative sex differences in eating behavior (e.g., boys exhibiting more food neophobia and had a lower body mass index (BMI) than girls). There is not a consensus if sex is associated with eating behavior problems in middle childhood [53]. A recent ASD population-based cohort study [54] highlighted how associations for eating disorders did not differ by child sex. This aspect should be better investigated with a bigger sample.

Despite these encouraging results, several limitations can be acknowledged. First of all, we did not investigate differences in the ASD population according to severity of symptoms, while emerging evidence suggests an overlap between the severity of ASD symptoms and eating problems, like food selectivity [41]. Second, we did not investigate the BAMBI score against other measures to verify the criterion validity that reflects the degree to which the scores of a measurement instrument are an adequate reflection of a gold standard [55]; moreover, a Receiver Operating Characteristics (ROC) curve was not calculated, though it should be extremely useful to determine the predictive power for this feeding measure. Considering the present limitations of the study, the results should be interpreted with caution; ASD implies a very heterogeneous group of people with different levels of functioning, while we considered all of them as a homogeneous group. Further studies should investigate differences across functioning, using a comprehensive evaluation of children.

Since food and, in general, mealtime is a convivial event with high social and cultural value, it would be interesting to assess the correlation that may exist between food selectivity and the development of communication and social skills of people with ASD, and how this characteristic is delineated in different cultures. Finally, it would be desirable to train rehabilitation professionals who can develop individualized programs and strategies that consider the specific difficulties of these children to reduce and extinguish food selectivity, thereby promoting the well-being of the affected individuals and the entire family unit.

In conclusion, the BAMBI has proven to be a reliable, quick, and easy-to-use tool and can be used for clinical and research purposes. The flexibility and specificity of the scale identified in this study also allow it to be used in various clinical and research settings depending on the purpose and participants.

## Figures and Tables

**Table 1:** Sample characteristics and BAMBI score.

	TD Sample (*n* = 84)		ASD Sample (*n* = 47)
Female *n* (%)	49 (58.3)		11 (23.04)
Mean (SD) age	6.73 (2.5)		7.45 (1.8)
Body Mass Index mean (SD)	25.76 (10.4)		24.89 (8.7)
**Siblings *n* (%)**			
0	22 (26.2)		39 (82.9)
1	46 (54.8)		8 (17.1)
2	14 (16.7)		0
3	2 (2.4)		0
**Gastric or eating disorders *n* (%)**			
Food allergy	2 (2.4)		9 (19.1)
Constipation	4 (4.8)		8 (17.0)
Gastroesophageal reflux	2 (2.4)		2 (4.2)
Abdominal tension	3 (3.6)		5 (10.8)
No	73 (86.9)		23 (48.9)
**BAMBI Score (total 131)**			
**Item**	**Mean (SD)**	**Problem No *n* (%)**	**Problem Yes *n* (%)**
1	1.33 (0.8)	73 (86.9)	11 (13.1)
2	1.52 (0.9)	71 (84.5)	13 (15.5)
3	4.05 (1.2)	68 (81)	16 (19)
4	1.33 (0.8)	72 (85.7)	12 (14.3)
5	1.10 (0.4)	77 (91.7)	7 (8.3)
6	1.00	78 (92.9)	6 (7.1)
7	1.29 (0.9)	76 (90.5)	8 (9.5)
8	1.48 (0.8)	75 (89.3)	9 (10.7)
9	4.67 (0.6)	82 (97.6)	2 (2.4)
10	3.67 (1.8)	66 (78.6)	18 (21.4)
11	2.86 (1.6)	65 (77.4)	19 (22.6)
12	1.14 (0.6)	79 (94)	5 (6)
13	2.10 (1.6)	73 (86.9)	11 (13.1)
14	1.71 (1.1)	81 (96.4)	3 (3.6)
15	4.10 (1.6)	72 (85.7)	12 (14.3)
16	1.43 (0.8)	83 (98.8)	1 (1.2)
17	1.38 (0.8)	75 (89.3)	9 (10.7)
18	1.57 (1.1)	79 (94)	5 (6)

TD—typical development; ASD—Autism Spectrum Disorder. BAMBI: Brief Autism Mealtime Behavior Inventory; SD Standard Deviation;

**Table 2:** Item-total statistics of BAMBI across groups.

Children with Typical Development Sub-Sample					
	Scale Means if Item Deleted	Scale Variance if Item Deleted	Corrected Item–Total Correlation	Squared Multiple Correlation	Cronbach’s Alpha if Item Deleted
ITEM1	27.90	79.991	0.307	0.437	0.834
ITEM2	27.92	78.077	0.589	0.607	0.824
ITEM3	27.23	74.996	0.388	0.314	0.832
ITEM4	27.98	80.867	0.289	0.420	0.834
ITEM5	28.21	84.074	0.245	0.487	0.837
ITEM7	28.02	79.542	0.428	0.606	0.829
ITEM8	27.83	75.635	0.586	0.435	0.821
ITEM9	27.32	81.353	0.101	0.214	0.849
ITEM10	26.99	66.301	0.745	0.794	0.806
ITEM11	25.99	70.952	0.503	0.367	0.825
ITEM12	28.00	81.976	0.214	0.372	0.837
ITEM13	27.13	68.718	0.686	0.576	0.811
ITEM14	27.36	77.558	0.334	0.325	0.833
ITEM15	27.15	69.313	0.643	0.757	0.814
ITEM16	27.69	78.505	0.380	0.475	0.830
ITEM17	27.73	76.008	0.486	0.533	0.825
ITEM18	27.55	72.684	0.639	0.579	0.816
**Children with Autism Spectrum Disorder Sub-Sample**					
	**Scale means if item deleted**	**Scale variance if item deleted**	**Corrected item–total correlation**	**Squared multiple correlation**	**Cronbach’s Alpha if item deleted**
ITEM1	34.52	84.477	0.319	0.790	0.703
ITEM2	34.15	79.554	0.527	0.725	0.683
ITEM3	33.43	90.162	0.006	0.403	0.733
ITEM4	34.65	83.165	0.527	0.807	0.691
ITEM5	34.93	87.618	0.345	0.796	0.706
ITEM7	35.04	91.687	0.092	0.548	0.718
ITEM8	34.67	86.491	0.277	0.672	0.707
ITEM9	34.26	77.442	0.660	0.695	0.671
ITEM10	33.76	89.742	−0.006	0.305	0.740
ITEM11	32.80	78.916	0.380	0.562	0.695
ITEM12	33.24	78.275	0.440	0.665	0.688
ITEM13	34.70	90.083	0.054	0.629	0.723
ITEM14	33.87	76.471	0.503	0.765	0.680
ITEM15	33.98	85.488	0.164	0.378	0.719
ITEM16	33.28	84.563	0.164	0.540	0.721
ITEM17	34.11	74.455	0.588	0.654	0.670
ITEM18	34.28	83.229	0.283	0.552	0.706

**Table 3:** Test–retest reliability analysis.

BAMBI	Mean (SD)	Mean (SD)	ICC 95% CI
ITEM1	1.33 (0.8)	1.43 (0.7)	0.91 [0.78–0.96]
ITEM2	1.52 (0.9)	1.43 (0.6)	0.85 [0.63–0.93]
ITEM3	4.05 (1.2)	4.14 (1.2)	0.96 [0.92–0.98]
ITEM4	1.33 (0.8)	1.24 (0.7)	0.96 [0.79–0.96]
ITEM5	1.10 (0.4)	1.05 (0.2)	0.83 [0.67–0.96]
ITEM6	1.00	1.00	1
ITEM7	1.29 (0.9)	1.19 (0.7)	0.91 [0.80–0.97]
ITEM8	1.48 (0.8)	1.48 (0.8)	1
ITEM9	4.67 (0.6)	4.67 (0.6)	1
ITEM10	3.67 (1.8)	3.67 (1.8)	1
ITEM11	2.86 (1.6)	2.86 (1.7)	0.99 [0.98–1]
ITEM12	1.14 (0.6)	1.14 (0.6)	1
ITEM13	2.10 (1.6)	2.10 (1.6)	1
ITEM14	1.71 (1.1)	1.67 (1.1)	0.99 [0.97–1]
ITEM15	4.10 (1.6)	4.05 (1.7)	0.99 [0.98–1]
ITEM16	1.43 (0.8)	1.33 (0.7)	0.96 [0.90–0.98]
ITEM17	1.38 (0.8)	1.52 (1.2)	0.94 [0.86–0.97]
ITEM18	1.57 (1.1)	1.52 (1.1)	0.99 [0.97–0.99]

BAMBI: Brief Autism Mealtime Behavior Inventory; SD Standard Deviation; Intraclass Correlation Coefficient; CI Confidence Interval.

**Figure A1:**
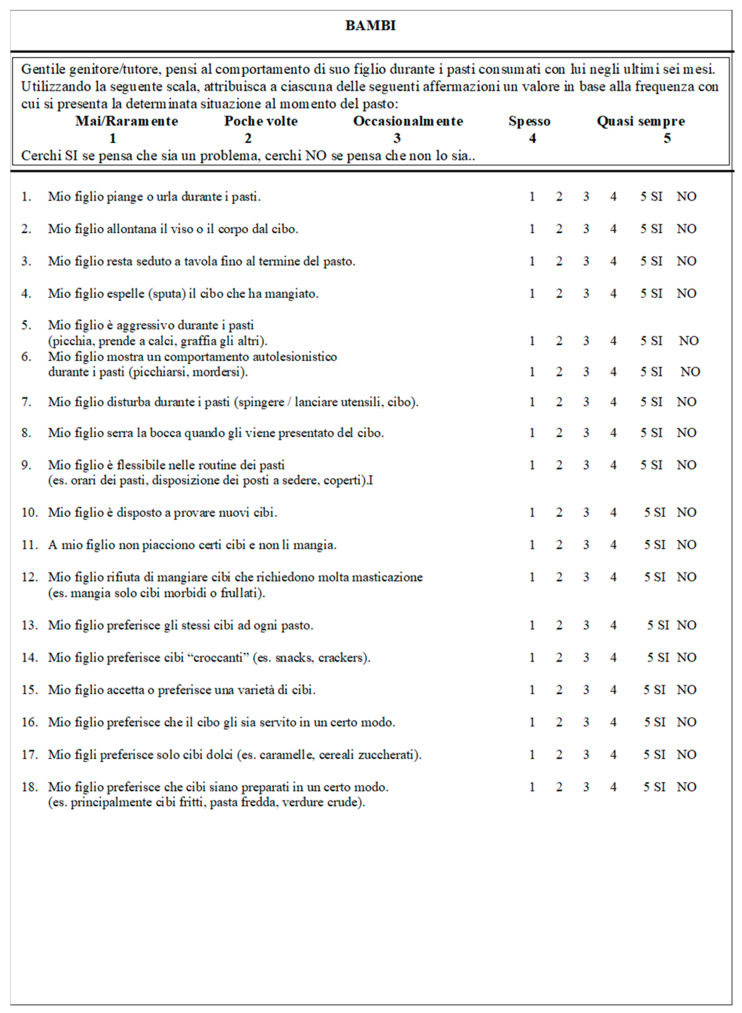
Italian Version of the Brief Autism Mealtime Behavior Inventory (BAMBI).

## Data Availability

Not applicable.
